# Patient-Tailored, Home-Based Non-invasive Brain Stimulation for Memory Deficits in Dementia Due to Alzheimer's Disease

**DOI:** 10.3389/fneur.2021.598135

**Published:** 2021-05-20

**Authors:** Lucie Bréchet, Wanting Yu, Maria Chiara Biagi, Giulio Ruffini, Margaret Gagnon, Brad Manor, Alvaro Pascual-Leone

**Affiliations:** ^1^Hinda and Arthur Marcus Institute for Aging Research and Center for Memory Health, Hebrew SeniorLife, Boston, MA, United States; ^2^Department of Neurology, Harvard Medical School, Boston, MA, United States; ^3^Neuroelectrics Barcelona, Barcelona, Spain; ^4^Neuroelectrics Corp., Cambridge, MA, United States; ^5^Department of Medicine, Harvard Medical School, Boston, MA, United States; ^6^Guttmann Brain Health Institute, Institut Guttman de Neurorehabilitació, Barcelona, Spain

**Keywords:** transcranial alternating current stimulation, Alzheimer's disease, memory improvement, home-based treatment, stimulation modeling, translational science, parietal cortex, angular gyrus

## Abstract

Alzheimer's disease (AD) is an irreversible, progressive brain disorder that can cause dementia (Alzheimer's disease-related dementia, ADRD) with growing cognitive disability and vast physical, emotional, and financial pressures not only on the patients but also on caregivers and families. Loss of memory is an early and very debilitating symptom in AD patients and a relevant predictor of disease progression. Data from rodents, as well as human studies, suggest that dysregulation of specific brain oscillations, particularly in the hippocampus, is linked to memory deficits. Animal and human studies demonstrate that non-invasive brain stimulation (NIBS) in the form of transcranial alternating current stimulation (tACS) allows to reliably and safely interact with ongoing oscillatory patterns in the brain in specific frequencies. We developed a protocol for patient-tailored home-based tACS with an instruction program to train a caregiver to deliver daily sessions of tACS that can be remotely monitored by the study team. We provide a discussion of the neurobiological rationale to modulate oscillations and a description of the study protocol. Data of two patients with ADRD who have completed this protocol illustrate the feasibility of the approach and provide pilot evidence on the safety of the remotely-monitored, caregiver-administered, home-based tACS intervention. These findings encourage the pursuit of a large, adequately powered, randomized controlled trial of home-based tACS for memory dysfunction in ADRD.

## Introduction

Memories fundamentally shape who we are. The loss of memory has a profound impact on the patient but also on spouses, family, and friends. Currently, there are neither effective interventions nor approved disease-modifying therapies that can effectively address memory deficits in patients with dementia. Cognitive science and neuroimaging studies have investigated memory extensively and identified a core network in the brain that supports memory function and comprises the hippocampus, medial prefrontal cortex, and angular gyrus (1–3). Activation of these structures seems crucial to retrieve the conscious experience of one-self in the past, and damage to this network can have profound consequences to everyday life (4). Consistently with the discovery of severely amnesic patients following damage to the medial temporal lobe (MTL) (5), many fMRI studies have established the essential role of MTL structures in memory (3, 6). Growing evidence from clinical studies of patients with lesions in the angular gyrus shows that the lateral parietal cortex is also critical for the subjective, conscious experience of retrieving vivid, vibrant, and multisensory (e.g., auditory and visual) episodic memories (7). A deeper understanding of the neurobiological substrate of memory could enable the development of early diagnostic indicators of dementia and novel therapeutic approaches to minimize cognitive disability.

We hypothesize that knowledge about the brain structures and brain network involved in memory encoding and retrieval is necessary but not sufficient to decode the underlying neurobiological mechanism. Specifically, we contend that brain oscillations, which arise from synchronized interactions between neuronal populations, are essential for memory processes. Previous electrophysiological studies have related episodic memory processes to specific oscillatory signatures. Theta oscillations (4–8 Hz) have been identified as the dominant rhythms recorded in the hippocampus, the critical structure for memory formation (8). It has been hypothesized that hippocampal theta not only serves as the binding mechanism for spatial navigation (place cells) but also for forming episodic memory, due to the overlapping nature of brain networks supporting navigation, remembering the past and thinking about the future (9). Apart from theta oscillations, gamma oscillations (25–100 Hz) have also been proposed as a prime candidate for memory-related synaptic changes in the hippocampus (10). A consistent finding in AD patients is a relative attenuation of gamma frequency (11) and dysregulation of gamma activity is also seen in animal models of AD (12). While theta and gamma oscillations in the hippocampus play a central role for episodic memory, the coupling between hippocampal theta activity and neocortical gamma oscillations seems to be the underlying mechanism that supports the interactions within the large-scale memory network (13). These studies provide essential insights into the roles of brain oscillations in memory impairment.

A recent seminal study in a mouse model of AD (5XFAD) found that optogenetic modulation of parvo-albumin positive (PV+) and somatostatin-expressing (SST+) interneurons can restore hippocampal network 40 Hz gamma oscillations and synaptic plasticity (14). Similar results could be induced by multisensory stimulation (15). Remarkably, induction of gamma oscillations via optogenetic activation or multisensory stimulation also modified inflammatory brain processes via activation of microglia and resulted in clearance of Aβ and p-tau deposition (14), and considerable cognitive benefits (15). Induction of gamma activity in presymptomatic AD mice even prevented subsequent neurodegeneration and behavioral deficits. This suggests that the induction of gamma oscillations may represent a novel and powerful therapeutic approach for AD. A translation of this work to humans seems feasible and promising.

Notably, it has been shown that in humans, transcranial Alternating Current Stimulation (tACS) can safely, and selectively enhance gamma or theta oscillations in specific brain regions (16). Others have also shown that modulation of brain oscillations in humans with other forms of transcranial Electric Stimulation (tES) is possible and may alter mechanisms of brain plasticity (17) and memory functions (18–25). tACS is a safe, non-invasive tES technique that utilizes low-amplitude alternating currents to modulate brain activity and entrain specific cortical rhythms depending on the applied stimulation frequency (26). The effects of tACS modulation of brain oscillations can outlast the duration of stimulation (27). tACS can be targeted to different cortical brain regions depending on the electrode montage. A recent method for optimizing the configuration of multifocal tACS for stimulation of specific brain networks (28, 29) has been developed. Such multifocal brain network targeting achieves a more significant behavioral and physiological impact than traditional tACS approaches using two large electrodes (30).

In a controlled, laboratory-based pilot study, patients with AD underwent 10 or 20 sessions of tACS at the gamma frequency, and brain magnetic resonance imaging (MRI), positron emission tomography (PET) for amyloid, tau or microglial activation, electroencephalography (EEG), and neuropsychological evaluations were completed before and after the intervention. Preliminary results reveal that (1) tACS can induce a significant induction of gamma activity in patients with AD that can be demonstrated on EEG; and (2) tACS-induced modulation of amyloid and tau deposition is demonstrable by PET, consistent with results in the murine models (ClinicalTrials NCT03412604). However, 10 or 20 sessions of stimulation are not enough to induce apparent behavioral and cognitive effects, and long, multi-week courses of laboratory-based interventions are challenging for patients with dementia and their caregivers (unpublished data).

In the present study, we aimed to test the feasibility and safety of a home-based, cost-effective stimulation system that could be used as an intensive neurorehabilitative treatment for long-term memory improvement in patients with dementia. Specifically, our aims were (1) Assess whether repeated, daily sessions of 40 Hz tACS can be applied safely and reliably at home by a caregiver following appropriate instruction; (2) Evaluate the tolerability, acceptability, and adherence of such a home-based program; (3) Gain very preliminary insights as to whether such a stimulation protocol can be run remotely and can lead to memory improvement in older cognitively impaired participants with ADRD. We hypothesized that a multi-week regimen of daily sessions of 40 Hz tACS to the left angular gyrus (AG) would be safe, well-tolerated, and adhered to, and would result in improvements in the memory performance. We selected the AG as the target area, based on previous studies (31–33) and based on the growing evidence that this brain area is critical for the subjective, conscious experience of retrieving vivid, rich and multi-sensory episodic memories (7, 34, 35). Importantly, we hypothesized that this small pilot would establish feasibility for a large-scale trial conducted at patients' homes with minimal disruption and high scalability.

## Materials and Methods

### Study Participants

#### ADRD Subjects

The ADRD subjects were both 79 years old men with an established diagnosis of dementia supported by clinical, neuropsychological, neuroimaging, and biomarker results. Both participants were on the same pharmacological regimen throughout the trial, and their medication regimen had been stable for at least 4 months prior to starting the study. Both were taking donepezil at a dose of 5 mg twice per day, and subject #1 was also on sertraline 25 mg once per day. Both participants had a typical amnesic phenotype with no prominent anomia. Both underwent a detailed neurological exam and medical evaluation and a comprehensive neuropsychological battery to characterize cognitive function. Baseline dementia severity was assessed using the Clinical Dementia Rating (CDR) scale (36) and the Montreal Cognitive Assessment (MoCA) (37). The participants' MoCA values using the standard version of the Montreal Cognitive Assessment which includes visual and other tasks, were 21 and 23 at the baseline. Despite the reported overall good correlation between visual, in-person MOCA and the 5 min, telehealth, non-visual MOCA, our two participants showed substantially lower scores in their non-visual MoCA scores (13 and 17, respectively) owing to their particular deficits in verbal memory, verbal word-list generation, and orientation. A cutoff of the MoCA score >18 for inclusion was applied. Both were willing and deemed capable of giving informed consent for participation in the study after the study was thoroughly explained. Understanding of the Informed Consent form was assessed by asking potential participants to answer the following questions: “What is the purpose of the study?”, “What are the risks of study involvement?” and “If you decide to participate, are you allowed to withdraw from the study at any time?”. Answers were recorded and reviewed for detail and correctness by the study physician. Insufficient understanding or unwillingness to participate would have led to exclusion from the study. Neither participant had any contraindication to tACS, as recorded on a standardized screening questionnaire, which included a history of seizures, use of neuroactive drugs, active dermatological condition (e.g., eczema on the scalp), or presence of specific implanted medical devices (e.g., deep brain stimulator, medication infusion pump, cochlear implant, or pacemaker).

#### tACS Administrators

The spouses of the two ADRD subjects participated in the study as “tACS administrators” and were taught how to administer home-based tACS. Both administrators self-reported computer proficiency and willingness to learn how to use tACS as defined by “yes” answers to the questions: “Do you feel comfortable using a computer?” and “Are you willing to learn how to administer tACS?”. Insufficient understanding or unwillingness to participate would have led to exclusion from the study. The administrators stated their availability during weekdays throughout the study period to administer tACS to the participants. Inclusion criteria included a MoCA score >26 during the in-person screen, sufficient understanding of study procedures following review of the Informed Consent form, and no health condition, such as poor eyesight, that could interfere with the successful administration of the tACS sessions. Both tACS administrators underwent a detailed neurological exam and medical evaluation and a comprehensive neuropsychological battery that ruled out dementia or other significant neurological or psychiatric disorders. Both were female (ages 78 and 73 years), had a CDR of 0, and a MoCA of >28/30 points. None of them had any contra-indication to tACS, as recorded on the same standardized screening questionnaire used in the patients.

### EEG Recordings and Analysis

The brain activity of all participants was recorded with high-density EEG during an autobiographical memory task at the beginning of the study. The EEG findings in the two study ADRD participants were compared with those in the two age-matched, cognitive-unimpaired tACS administrators who underwent the same EEG procedure as part of a parallel study protocol approved by the institutional review board.

EEG was recorded with a 256-channel EGI system (Electrical Geodesic Inc., OR, USA), a sampling rate of 1,000 Hz and Cz as acquisition reference. All EEG analysis was performed using the freely available academic software Cartool developed in the Biomedical Imaging Centre Geneva-Lausanne (38) (https://sites.google.com/site/cartoolcommunity/). The EEG was down-sampled offline to 250 Hz; bad electrodes were interpolated using a 3D spherical spline, bandpass filtered between 1 and 100 Hz, and the data were re-referenced to the common average-reference. Infomax-based Independent Component Analysis (ICA) was applied to remove oculomotor and cardiac artifacts.

**Figure 1:**
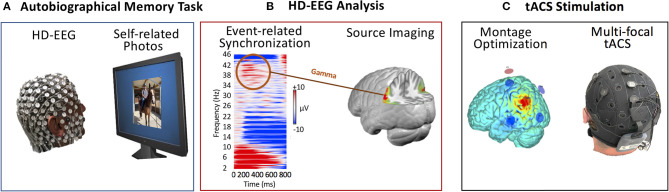
Roadmap of EEG-guided personal tACS to improve autobiographical memory. **(A)** High-density EEG recording during a personalized autobiographical memory task that requests to decide whether pictures are self-related or not. **(B)** Analysis of the high-density EEG in terms of stimulus-evoked frequency modulations. Determination of the individual peak frequency of event-related synchronization and source localization of this frequency based on individual structural MRI head models. **(C)** tACS montage optimization based on the EEG result to perform multi-focal stimulation at the individual gamma peak frequency targeting the sites determined by the EEG source imaging.

The EEG was analyzed using time-frequency decomposition to uncover the stimulus-induced synchronization/desynchronization after the presentation of self-relevant pictures and the differences of these spectral changes between controls and the two ADRD subjects. To determine the sources of the stimulus-induced frequency changes, the signal was bandpass filtered in the frequency band of interest. A distributed linear inverse solution (LAURA) (39) was calculated using the average brain of the Montreal Neurological Institute as the head model and restricting the solution space to the gray matter using the LSMAC approach with 5,016 solution points (40). Figure 1 depicts the roadmap to improve autobiographical memory (Figure 1A) using EEG-guided (Figure 1B), personal tACS stimulation (Figure 1C).

### tACS Administration

tACS was delivered by a wireless, battery-driven current stimulator (Starstim SS32, Neuroelectrics), through surface Ag/AgCl electrodes (1 cm^2^ radius circular surface contact area) placed into holes of a neoprene cap corresponding to the international 10/20 EEG system, with the Cz position aligned to the vertex of the head. Gel (Signa Gel, Parker Lab, Inc.) was applied to optimize signal conductivity and lower the impedance. Starstim resembles a swimming cap with small electrodes that lie on the scalp and deliver low-level electrical currents to targeted brain regions. Care was taken to ensure that the caregivers unambiguously placed the color- and number-coded electrodes in the correct position and secured the correct lead to each electrode. The Home Kit included a WIFI-enabled tablet running Starstim control software that provided the caregivers with step-by-step instructions to set up the Starstim device, check electrode impedance, and record side effects. The participants and the caregivers were able to videoconference with the remote staff in real-time. We used Neuroelectrics online Portal (**NE Portal**) to remotely schedule sessions and monitor in real-time specific treatment events to ensure safety.

### Feasibility, Safety, and Tolerability

The online NE portal provided information related to electrode impedance, tACS progress if the tACS session was aborted and, if so, then why it was aborted. Following each tACS session, the participants receiving tACS reported side effects and complications via a series of multiple-choice and open-ended questions on the tablet. This information was immediately available to the researcher. These metrics were used to assess feasibility, tolerability, and safety: the number of aborted sessions, the number of missed sessions. The tACS device contains a safety block such that only one tACS session can be administered within 24 h.

### tACS Montage Optimization

**Figure 2:**
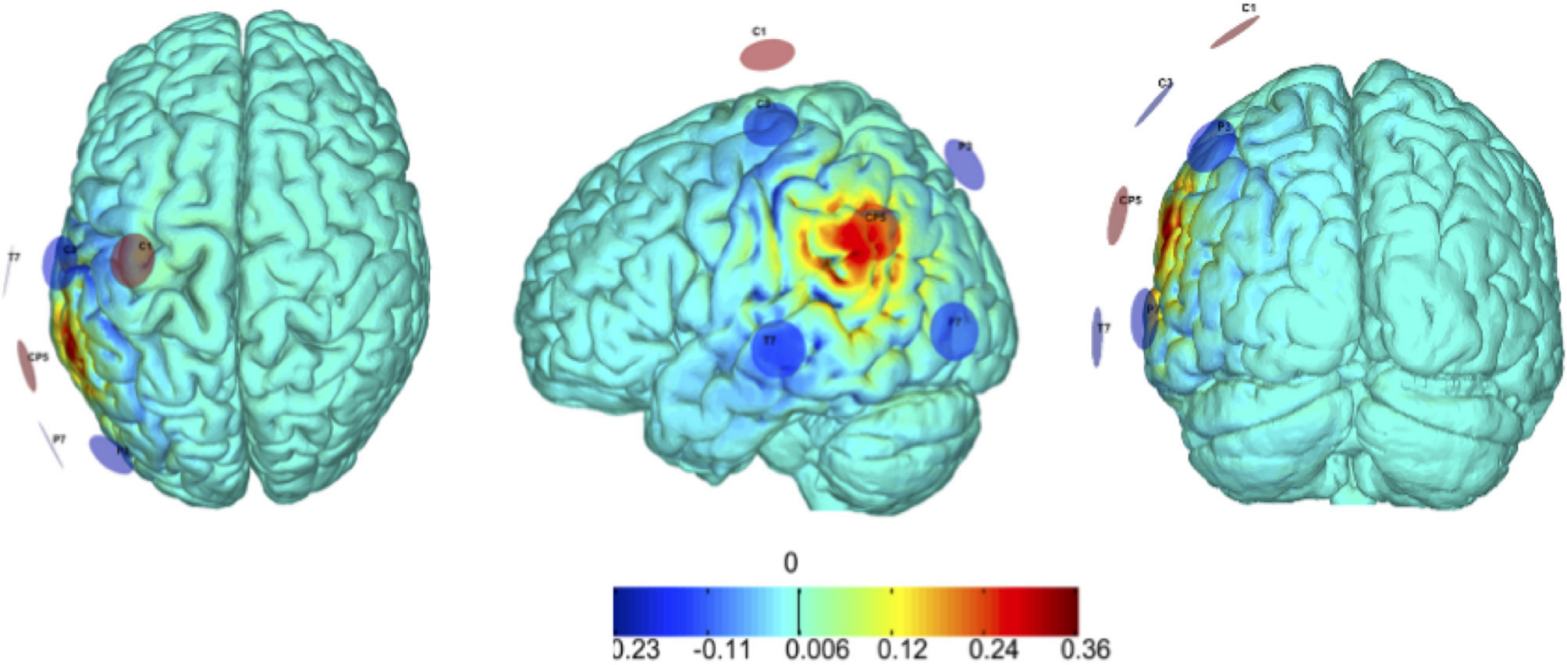
Modeling of electric field induced by tACS. tACS was delivered to the target region (left angular gyrus, BA39/40). Our montage optimization is based on the *StimWeaver* algorithm (29) that revealed that 6 stimulating channels, 2 anodes, and 4 cathodes, provide an optimal montage solution, with the precise stimulation pattern defined using modeling of the induced electric field. By design, the number of active electrodes depended on the optimization but was predefined to be kept always ≤ 8. In keeping with safety guidelines, the max total injected current was set to be < 4.0 mA and a maximum current per electrode was set to be < 2.0 mA.

Based on previous studies (31–33), we selected the left AG as the target area and a stimulation frequency of 40 Hz (gamma range). The AG target area was identified on a standard reference brain, which was also used to determine the optimized montage. In future studies, we suggest the use of a personalized head model for each patient to determine individually optimized montages. Personalized head models can be reconstructed from the individual MRIs of each specific participant, and the target areas should be mapped onto each cortex after registration in common reference space [see, e.g., (41)]. The montage optimization study was performed using the *Stimweaver* algorithm (29), which revealed an optimized solution for the target area (the left AG, BA39/40) with a montage of 6-electrodes in standard EEG electrode positions (circular electrodes with a contact area of 3.14 cm^2^). The Stimweaver algorithm does not use fixed assignment of certain electrodes as cathodes and others as anodes based on a pre-established arrangement. This is distinctly different from other multi-electrode approaches like the HD-stim where the central electrode can be the cathode and the surrounding electrodes are the anodes, or vice-versa. Applying this algorithm, we designed montage with 6 active electrodes, 2 anodes and 4 cathodes which induced maximal current in the left AG whilst minimized current induced in the rest of the brain outside the target area. The optimization of the tACS montage was designed to maximize on target the impact of the cortically standard component of the electric field, believed to be the main driver for the effects of tES (29), while avoiding stimulating more extensive off-target areas. The modeling fit quality of the 6-electrode montage was almost 99% of the one allowing for the maximal number of electrodes in the used cap (39 electrodes)—see Figure 2. Both participants received the same, well-controlled, and fixed montage (electrode locations and currents). During each session, a maximum total injected current of 4 mA was applied with a maximal current at any one electrode of 2 mA. The current intensity was ramped up over 30 s, then sustained at the stimulation intensity for 20 min, then ramped down over 30 s. A recent study by Reinhart and Nguyen (20) applied single session of tACS for 25 min in older adults and showed an increase in neural synchronization patterns within the frontotemporal brain regions. As a result, they reported a significant improvement in working memory. Therefore, we expected that 20 min would be sufficient to provide a significant effect, but future studies may want to consider longer stimulation sessions. For example, Iaccarino et al. (14) and Martorell et al. (42) applied 1 h stimulation sessions.

### Home-Based tACS Training for Administrators

The administrators were trained on how to use the tACS home equipment during a lab-based visit. They received a step-by-step overview of the home-based stimulation kit and completed several training sessions, including tACS administration and equipment care. The administrators then practiced setting up the tACS session on the ADRD participants who received stimulation, with oversight and coaching from the study staff. Study staff initiated a competency checklist (including electrode and machine preparation, stimulation) and identified specific areas that required more focused training. At the end of the training, we assessed and confirmed that the administrators achieved proficiency in applying the home-based tACS against a checklist of procedures confirming the ability to prepare the equipment independently and to operate the device safely. Administrators demonstrated competency in all procedures and self-reported comfort in doing so.

A pre-configured tACS system, including the device, tablet, all needed supplies, and training material was provided to the each of the ADRD participant-administrator pair. The tACS tablet contained a sequence of simplified instructions, and step-by-step touchscreen prompts for the participant/administrator to follow. This process was designed to be “older-adult-friendly” and easy to use, even if the administrators were not advanced computer users. A companion paper-based manual was delivered to the participants together with the tablet instructions. The trained administrators then applied 20 min-long tACS sessions in the morning to the participants at home for 14 weeks, 5 days per week, for a total of 70 sessions per participant. During the tACS sessions, participants were seated comfortably and remained at rest, stayed awake, and refrained from talking. The administrators were instructed to observe the participants, and if necessary, to encourage them to stay awake during each tACS session. In the beginning and at the end of each tACS session, participants completed a questionnaire to report potential side effects (e.g., skin irritation, headache, changes in alertness etc.).

### Cognitive Evaluation of the Participants

Since the evaluations were done remotely, we used the Mini/5 min, non-visual version of the MoCA (37, 43) to assess the cognitive effects of the intervention bi-weekly. Three different versions of the MoCA were employed to minimize the risk of carry-over and learning effects. The non-visual MoCA scores of the two ADRD subjects were 13 and 17 in the beginning of the study and 29 and 21 at the end of 14 weeks of home-based tACS stimulation. Given the focus and aims of the study, we focused on the Memory Index Score as defined and adopted in the Uniform data set of the National Alzheimer's Coordinating Center. The Memory Index Score is based on the delayed recall of 5 words and awards 3 points for each word that is freely recalled without a cue for a maximal score of 15. Thereafter, cues were offered only for words not recalled spontaneously. Two points were awarded for each word recalled with a categorical cue (e.g., “one of the words was a color”). Finally, 1 point was awarded for each of the still missing words correctly identified on recognition (e.g., “was one of the words daisy, rose, or tulip”). Both participants were tested every 4 weeks after the completion of the study for potential carryover effects.

## Results

Our preliminary results document the feasibility of the approach, support the safety of the intervention, and provide essential information regarding the potential beneficial effects of tACS on cognitive function in ADRD participants.

### EEG Results

**Figure 3:**
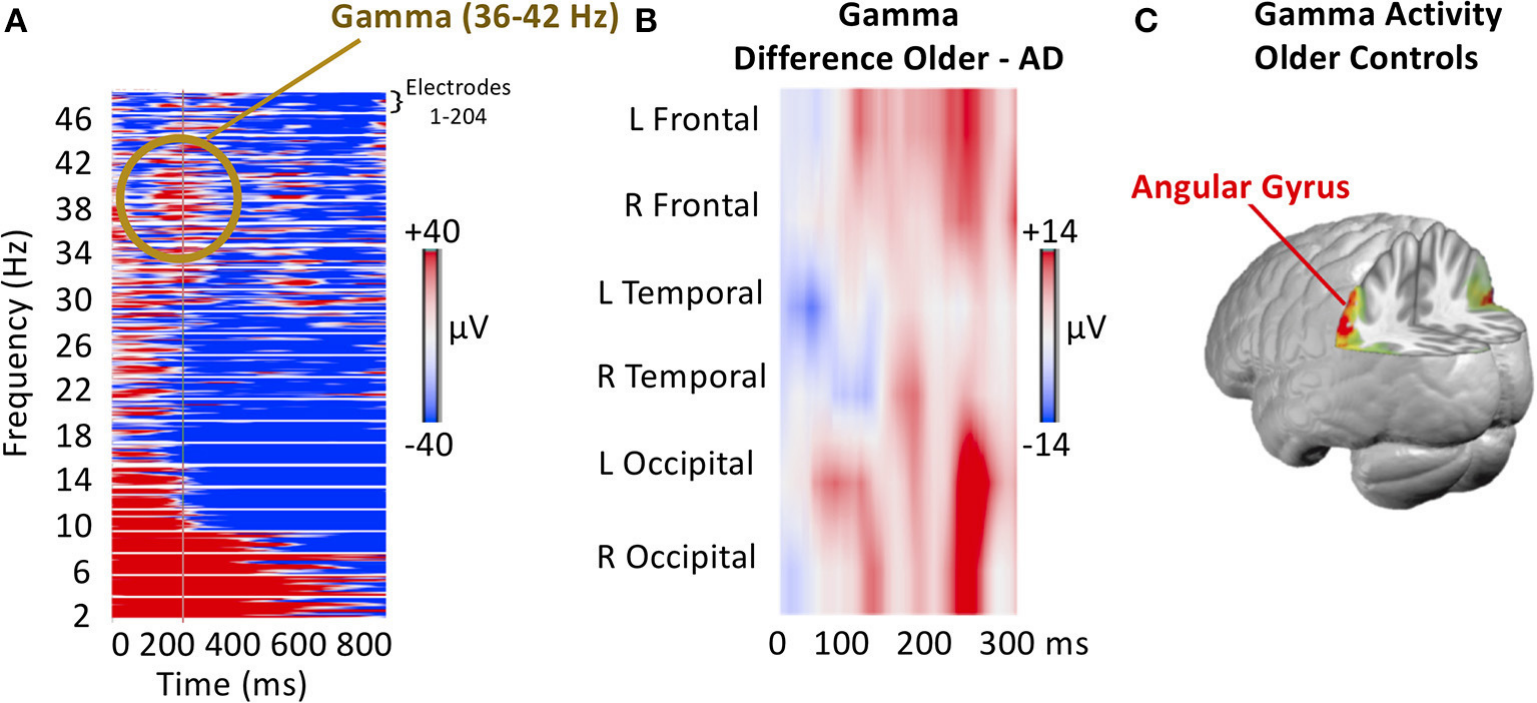
EEG Frequency modulation induced by the presentation of autobiographical photographs. **(A)** Example of induced power changes from 2 to 44 Hz after subjects responded, indicating having recognized a picture as self-related. Each line represents the 257 electrodes in the given frequency. Note the power increase in theta and gamma and the decrease in alpha. **(B)** The induced gamma power difference between participants with ADRD and age-matched cognitively-unimpaired controls. Electrodes are averaged for regions of interest, as indicated. Note the more considerable gamma power increase for recognized pictures in controls as compared to ADRD participants. **(C)** Source localization of task-related gamma activity increase averaged across the period of 100–300 ms after stimulus onset. Note the left inferior parietal activity in the region of the angular gyrus.

The results of the high-density (hd) EEG data using time-frequency analysis (Figure 3) are consistent with the literature: the memory task-induced frequency power increases in the theta (2–8 Hz) and the gamma range (36–42 Hz) and power decrease in the alpha and beta range (10–30 Hz) (Figure 3A). No stimulus-induced changes were observed in the high gamma range. The memory stimuli-induced increases in theta and gamma oscillations were reduced in the two AD patients as compared to nine cognitively-unimpaired controls (6 females, age range 66 ± 8 years) (Figure 3B). Source localization of the gamma oscillations (36–42 Hz bandpass filtered EEG) revealed generators in the inferior parietal lobule (Brodmann Area 39) (Figure 3C).

### Adherence, Safety Monitoring, and Risk Assessments

Both participant-administrator pairs showed perfect adherence to the protocol (100%), missing none of the 70 scheduled sessions in the 14 study weeks. The researchers did not have to intervene to resolve any major technical difficulties. In total, the sessions were twice auto-aborted due to high impedance. It is noteworthy that the lab-based training, including an assessment of competency which the administrators needed to pass prior to starting the study, prepared the administrators for such situations and therefore they were able to independently complete the stimulation sessions. The home-based stimulation sessions were remotely supervised by the researchers who received information about impedance, tACS progress, and, potentially, session aborts or interruptions in real-time. The participants and administrators could initiate a video conference before, during, or after the stimulation sessions and immediately communicate or report any issue, if necessary, to the researchers. The study was designed to minimize risks to the study participants. The tACS was ramped up and down to reduce the occurrence of unpleasant sensations under the electrode. The participants were monitored throughout the stimulation period and were evaluated by a researcher each day before and after the stimulation. All home stimulation sessions were, therefore, supervised.

### Report on Tolerability, Side Effects, and Technical Difficulties

Participants completed self-report measures to address tolerability before and after each session. Reported side effects were the expected (44), common side-effects of tACS and were all mild. All the side effects resolved after the completion of the stimulation session. Side effects mainly included sensations of tingling and burning on the electrode site. There were no skin lesions. There were few occurrences of headache, and one occurrence of difficulty concentrating following a session. Reported technical difficulties during the home-based sessions were common of tACS and mild. Technical issues mainly included measures of high electrode contact impedance, which resolved after adding extra gel under the specific electrodes. There were few occurrences of session interruptions and aborts due to high-impendence and due to internet connection cutoffs. In all instances, it was possible to address the issue and resume the session with minimal delay. None of them led to the missed sessions (all sessions were completed).

### Cognitive Effects

#### Performance in Formal Testing

**Figure 4:**
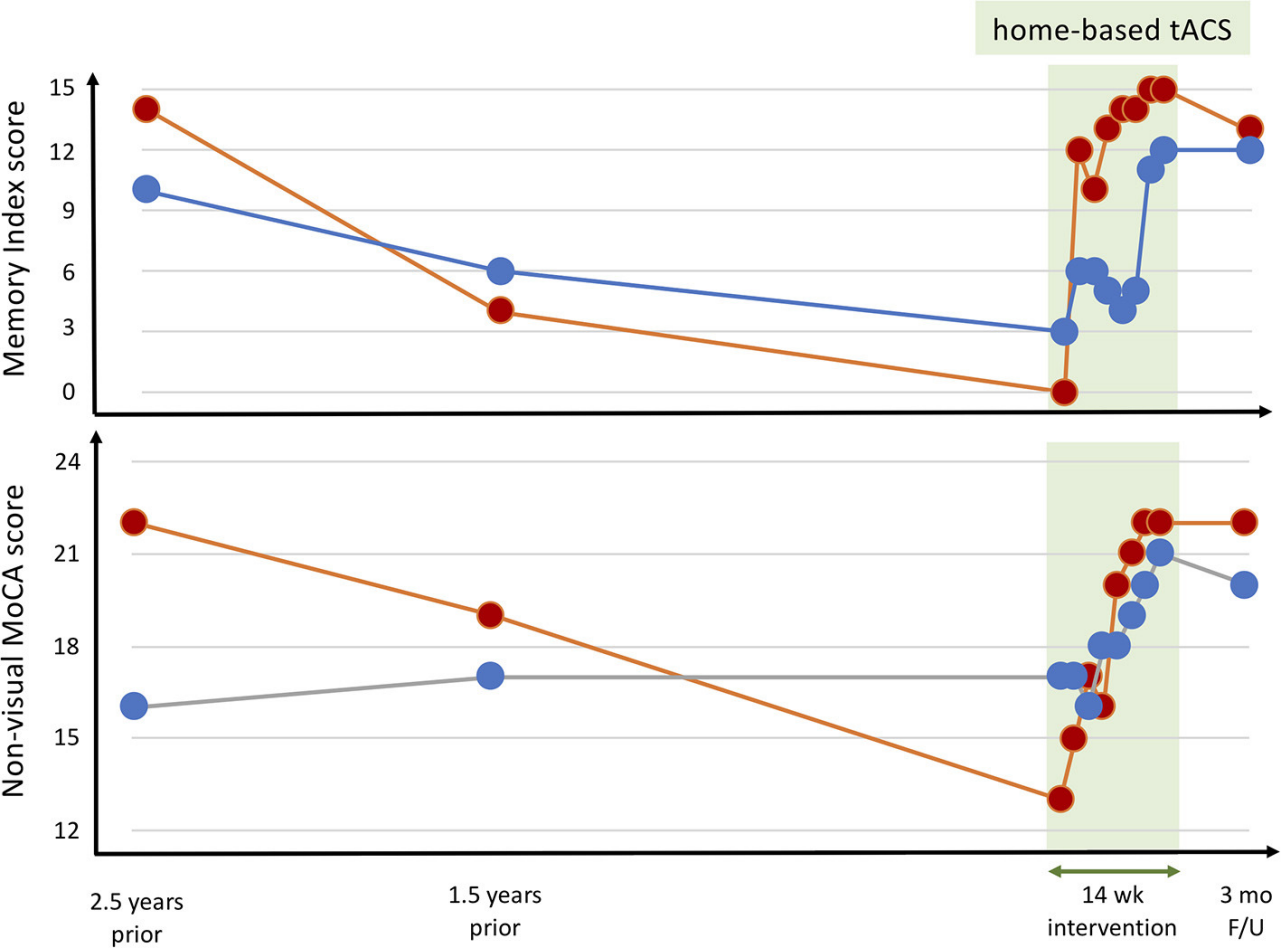
Cognitive performance throughout a 14-week intervention of open-label, home-based, caregiver-administered tACS in two ADRD participants. Both participants completed all scheduled stimulation sessions and tolerated the intervention with no report of adverse events or complications. The Mini/5 min non-visual versions of the Montreal Cognitive Assessment (MOCA) were evaluated via telehealth rotating between three different versions to avoid learning or carry-over effects. The equivalent metrics from the standard MOCA were extracted from the neuropsychological evaluations completed on both participants 2.5 and 1.5 years before study participation. By definition, the control subjects had normal memory index scores (a score of 14 or 15 in all cases) and normal MOCA scores (a score of 27–30 in all cases).

Over the 14 weeks of the study, both participants exhibited an improvement in the testing completed every 2 weeks as compared with baseline (Figure 4). These data are uncontrolled and on only two participants and should, therefore, be considered extremely preliminary. Nonetheless, the study provides clear, initial evidence that home-based, remotely supervised tACS treatment is feasible for participants with memory deficits and offers a potentially effective intervention.

In the Memory Index assessment, at baseline, one of the participants (Figure 4, red) was not able to recall any of the words, not even after categorical or multiple-choice cues, and in fact, did not also recall having been given any words to remember. At the end of the 14 weeks, however, he correctly freely recalled all 5 words. The other participant at baseline was only able to identify three of the five words from the multiple-choice cue, but at the end of the tACS course, he freely recalled three of the words, recalled the fourth with a categorical cue and correctly identified the last one from multiple-choice options. Of note, that participant had obstructive sleep apnea, and midway through the tACS course had his CPAP device exchanged because of concerns that it was not working correctly. Following the exchange of the CPAP device, the participant reported feeling more rested in the morning and having a “clearer head,” and the Memory Index score improved substantially (from 5 to 11).

#### Qualitative Report of the Impact of Daily Activities

Both participant pairs were queried bi-weekly on their qualitative impressions regarding cognitive and memory performance. The spouses reported noticing definite improvements in the daily activities of their husbands but also commented on fluctuations from day to day. Both commented, though, on increasing number of instances where they were surprised, their husbands recalled a given piece of information or event. Overall they both felt there were fewer instances of repeating asking of the same questions, better orientation, greater recall of plans, etc. For example, one of the participants and his wife play bridge, and the wife commented that the ADRD participant's play improved substantially, with his being able to follow the games better, recall conventions, and overall regaining a level of play he seemed to have lost. At another time, the wife noted that he recalled not only playing golf the day before but also with whom and what the scores had been.

## Discussion

This study provides preliminary data to support the feasibility, safety, and home-based tACS in the ADRD population and encourages future research to increase access of tACS as part of memory decline prevention strategies and treatment for older adults with memory problems. Our data demonstrate that caregivers can be trained to apply home-based tACS competently and that tele-monitored tACS in participants with ADRD is well-tolerated and safe even in repeated consecutive daily sessions over many weeks. Furthermore, even though the data have to be taken with great caution given the uncontrolled nature of the intervention and the very small N, our data suggest that in participants with ADRD, tACS can induce clinically meaningful cognitive effects.

The EEG and the tACS results of this pilot study suggest that the left angular gyrus may be a suitable target brain area of the memory network for spatially-specific modulation of brain oscillations to induce memory enhancement. The angular gyrus has been recently shown to be involved in episodic, self-relevant memories (31–33), which is one of the earliest AD memory deficits. Patients with posterior parietal damage exhibit impaired free recall of autobiographical memories, however when cued, their memories seem to be intact (45). Even if the memory accuracy appears to be unaffected by the parietal lesions, the vividness, richness and subjective confidence in the personal memories is diminished (46, 47). Due to its specific cognitive functionality, we chose the left AG as the target region for brain stimulation. The targeted area is strongly associated with the internally directed processes, such as mind-wandering or thinking about one's past and future. The AG has been shown to activate during retrieval of episodic autobiographical memories. Imaging studies on mind-wandering have consistently shown activation in angular gyrus associated with the DMN. Interestingly, the disruption of the DMN in AD has been reported (48, 49). Our results thus have profound translational potential as tACS intervention is safe, portable, does not require expensive hardware, and can be widely applied to large numbers of MCI/ADRD participants as well as adults at earlier stages of other, related neurodegenerative diseases.

Histopathologically, AD is characterized by diffuse amyloid-β (Aβ) plaques and phosphorylated tau (p-tau) deposition in neurofibrillary tangles, as well as widespread neurodegeneration. PET imaging studies suggest that progressive Aβ deposition can begin decades before the onset of clinical symptoms and stabilizes around the time that clinical symptoms become prominent (50, 51). P-tau accumulates initially in the meso-temporal lobes and spreads outside the temporal lobes linked with Aβ. Neurodegeneration and clinical symptoms seem strongly correlated with the spread of p-tau (52). Memory network disfunction is an early, primary driver of cognitive disability and disease progression in dementia (53, 54). Recent studies demonstrate that in AD, even before the accumulation of Aβ and p-tau, Aβ oligomers cause synapse-specific dysfunctions in parvalbumin-positive (PV+) and somatostatin-positive (SST+) interneurons leading to a dysregulation of theta and gamma oscillations. Loss of gamma activity has been linked to alterations in the mechanisms of brain plasticity (55–57), an early finding in AD also thought to be related to the synaptic toxicity of Aβ oligomers (58, 59). Disrupted theta and gamma oscillatory activity is reflective of altered functional and structural brain integrity (60) and heralds p-tau and Aβ accumulation, memory deficits, and cognitive decline in AD (61).

Recent lab-based studies suggest the feasibility of interacting with brain oscillations using tACS. Due to its safety (44) and controllability (in terms of stimulation frequency and the possibility to target specific cortical regions), tACS appears to be a most promising technique to modulate activity in the healthy and pathological brain. Animal work has demonstrated that tACS can entrain neurons in widespread cortical areas (62) with the effects constrained by the individual endogenous cortical oscillations (26, 63–65). Simulations (26, 64, 66), supported by empirical evidence using EEG, demonstrate that tACS modulates oscillatory brain activity via network resonance, suggesting that a weak stimulation at a resonant frequency can cause large-scale modulation of network activity (67), and amplify endogenous network oscillations in a frequency-specific manner (26). In humans, tACS modulates brain activity, with effects being documented at the behavioral level for sensorimotor (68, 69), visual (70), somatosensory (71) and higher-order cognitive domains (19, 72), and lasting beyond the duration of stimulation (27). However, tACS interventions despite their safety are currently not accessible to large numbers of older adults who live in remote settings or who are unable to utilize and organize transportation, because such interventions must be administered over multiple in-person visits to a clinical or laboratory setting. This barrier hinders the ability of healthcare professionals to deliver tACS to those who stand to benefit the most. It also places a sizeable travel-related burden on clinical trials and thus slows the pace of discovery and technology transfer of tACS to vulnerable older adult populations. Our study addresses this need by demonstrating the feasibility of a remotely supervised and remotely controlled tACS in memory-related disorders and home-based settings.

Our primary goal was to demonstrate the feasibility of remotely-monitored, caregiver (or spouse)-administered, home-based tACS intervention to improve memory in older adults by modifying gamma activity. To do so, we used the Starstim Home Kit® (Neuroelectrics Corp). The Starstim Home Kit enabled us to “prescribe” home-based tACS to end-users. Following in-person training sessions in the lab, two ADRD participants and their caregivers took home a pre-configured and personalized Starstim system. The researcher monitored treatment progress remotely in real-time. Our preliminary findings reveal the promising nature of tACS as a paradigm for participants where repeated visits to the clinic are not feasible and provide the first evidence of adherence to multi-week, home-based tACS memory treatment. It is noteworthy that the current protocol is established in such a way that after the initial lab-based training, the healthy caregiver is able to run the home-based sessions independently and without continuous contact with the researcher. Participation from home, while still maintaining clinical trial standards and high-quality research, can resolve accessibility barriers and improve recruitment, thus enabling the implementation of more substantial and longer-lasting clinical trials. Our approach opens a new avenue for several innovative methodological features, including (1) a personalized multi-electrode montage will enable modulation of specific brain regions (29) (2) advanced modeling will provide insights into the tACS dose induced in the targeted brain areas (73).

### Limitations and Future Directions

We limited the EEG analysis on event-related spectral perturbation. Potentially interesting analysis would be of the ratio between high and low frequencies, as 1/f noise has been shown to increase with age (74). This 1/f analysis and its relation to clinical outcomes would be ideal to include in future studies. Future studies should also explore how the effects of tACS affect verbal vs. non-verbal memory, auditory procession, attention or lexical retrieval. This could be done by including diverse tests, specific for each category. Even though the clinical evaluation was performed every 2 weeks during the 14 weeks of the study and then every 4 weeks after the completion of the study, we cannot exclude a learning effect of the actual task. Therefore, future studies should include a diversity of cognitive tasks and evaluations. Given our design, we cannot be certain that the effects of daily home-based stimulation are specific to the gamma frequency. Therefore, future studies should consider appropriate controls for example in a parallel or cross-over trial design, which might ideally include sham stimulation sessions as well as tACS at other stimulation frequencies. Along the same lines, including a control condition of the target area would be important to evaluate whether targeting the angular gyrus is indeed critical. Future studies should also examine and thoroughly test the effects of stimulation based on participant's circadian rhythm. We found a carryover effect lasting up to 3 months after the end of the study. Future studies should examine further the longevity of such effects. Finally, it would be profitable for future studies to focus on biomarker+ AD (either positive amyloid PET scans or CSF) and gather structural and functional MRI scans at the beginning of the study and compare such markers at the end of the study.

### Multi-Electrode tACS for Precise Targeting

The spatial resolution of transcranial Electric Stimulation (tES) in general, and tACS, in particular, is limited. However, new technologies to increase the precision of tES targeting have been recently developed, allowing for individualized stimulation solutions based on modeling of current distribution using brain MRI scans. Commonly used tES devices only allow for stimulation protocols using two electrodes, limiting control of the target regions. Moreover, this does not allow for careful mapping of individual brain anatomy, which might be even more critical in the case of individuals with AD, where significant levels of cortical atrophy are expected, which might dramatically affect the induced electrical field. Multi-focal (i.e., multi-electrode) stimulation (29) permits montages with up to 32 stimulating channels, with the stimulation pattern defined using modeling of the induced electric field based on MRI scans. This approach allows for individualized montages to target specific brain networks. Given the cortical atrophy expected in patients with AD, individual tES models based on structural MRI will be necessary also to account for increased scalp-cortex distance and CSF volume, which can significantly alter the distribution of the induced electric field (75–77). Furthermore, current research efforts are being directed not only to more realistic modeling of the physics of tES and its associated electrical field but to its physiological impact. Such *hybrid brain models* integrate physics and physiology using neural mass models assembled with structural and physiological data (MRI, DTI, EEG). This framework will allow for more realistic and informed tES targeting and optimization strategies (78).

### Home-Based, Remote Technology-Supported Cognitive Assessment

The recent shut-down due to the world-wide pandemic has challenged the traditional model of running research studies in a laboratory environment. Since it seems feasible and safe to monitor a caregiver to administer home-based tACS interventions remotely, our results emphasize the need for reliable, validated remote, technology-supported cognitive assessment and digital measurements. We completed the present study conducting the MoCA using telehealth. However, more sensitive, remote technology-supported evaluations are needed. Critically, most of the neuropsychological assessments of memory are often disconnected from the personal, self-related events that AD participants experience in everyday life. Therefore, testing autobiographical memory in a more naturalistic, ecological way rather than using laboratory-based tasks, which are mostly self-irrelevant, would be beneficial for the AD participants. The traditional Clock Drawing Test (79) is a well-established and widely used pen and paper cognitive assessment in which participants draw the face of a clock showing a specific time on a blank page, then copy a pre-drawn clock. However, the DCTclock^TM^ online test uses new advances in technology to capture and analyze the small movements of drawing behavior remotely (80, 81) and provides more sensitive metrics that are correlated with tau and amyloid loads on PET in patients with AD (82). Such methods of evaluation would be ideal to combine with the home-based tACS sessions. Similarly, E-Prime go (https://pstnet.com/using-e-prime-for-remote-data-collection/) is a psychology software tool that enables to send experiments to participants at their homes and thus collect data remotely. The MoCA assessment itself is available as an online app and has been recently validated (83, 84). BrainCheck (https://braincheck.com) and others offer suitable, FDA-cleared computer-based cognitive assessments that can be acquired remotely. Linus Health (https://linus.health) captured multimodal signals, not only performance on neuropsychological tasks (including the DCTclock^TM^) but also voice, eye movement, and gait and balance metrics to provide a more holistic and ecologically valid measure of cognitive and behavioral function. These online platforms for remote data collection and assessment seem to be extremely promising.

### Conclusion

To our knowledge, this is the first remotely supervised tACS protocol designed explicitly for ADRD participants. The results of our exploratory study encourage more extensive, randomized sham-controlled studies of the therapeutic potential of gamma tACS over the left angular gyrus in ADRD. The possibility of effectively delivering tACS neuromodulation in home-based environments in a standardized, supervised fashion opens stimulating perspectives for the definition of new paradigms of neurorehabilitation and the development of well-powered, well-designed clinical trials.

## Data Availability Statement

The raw data supporting the conclusions of this article will be made available by the authors to qualified parties upon written request and consideration.

## Ethics Statement

The study was approved by the Institutional Review Board at Hebrew SeniorLife and conducted according to principles outlined by the Declaration of Helsinki and the International Ethical Guidelines for Biomedical Research Involving Human Subjects. Two pairs of participants, a subject with ADRD and prominent memory deficits and a caregiver capable and willing to learn and then apply the home-based tACS sessions, were recruited into this pilot study by the research study team following appropriate screening, which included detailed description of the study protocol, tACS eligibility screening, computer proficiency screening (see more details in section study participants), and written informed consent. These two pairs were the first two recruited into an ongoing study. The patients/participants provided their written informed consent to participate in this study.

## Author Contributions

LB, WY, MB, GR, MG, BM, and AP-L: conceptualization. LB, WY, MB, GR, and AP-L: methodology. MB, GR, and AP-L: software. LB, MB, GR, and AP-L: formal analysis and visualization. LB and AP-L: data curation and writing—original draft preparation. LB, MB, GR, BM, and AP-L: writing—review and editing. All authors contributed to the article and approved the submitted version.

## Conflict of Interest

AP-L is a co-founder of Linus Health and TI Solutions AG; serves on the scientific advisory boards for Starlab Neuroscience, Neuroelectrics, Magstim Inc., and MedRhythms; and is listed as an inventor on several issued and pending patents on the real-time integration of non-invasive brain stimulation with electroencephalography and magnetic resonance imaging. MB and GR were employed by Neuroelectrics. The remaining authors declare that the research was conducted in the absence of any commercial or financial relationships that could be construed as a potential conflict of interest.
